# Some Remarks on the Preservation and Management of Leeches

**Published:** 1804-12-01

**Authors:** G. Wilkinson

**Affiliations:** Member of the Medical Society of London, of the Royal College of Surgeons of Edinburgh, and Honorary Member of the Medical Society of Aberdeen, Literary and Philosophical Society of Newcastle upon Tyne, and Chirurgo-Physical Society of Edinburgh


### SOME REMARKS ON THE PRESERVATION AND MANAGEMENT OF LEECHES; *by* G. WILKINSON, *Member of the Medical Society of London, of the Royal College of Surgeons of Edinburgh, and Honorary Member of the Medical Society of Aberdeen, Literary and Philosophical Society of Newcastle upon Tyne, and Chirurgo-Physical Society of Edinburgh.*

GREATLY as the Art of Medicine has of late years been improved by the industry and diligence of its professors; yet so much remains to be done, that we can scarcely flatter ourselves (as human beings) that it will ever attain its *ne plus ultra*, or state of perfection. Hence it follows, that whatever tends to its advancement in the smallest degree, however frivolous it may appear to the indolent,

 careless, careless, and unreflecting part of mankind, will nevertheless be regarded as important by the wise and prudent.

I have been led to these reflections from the inquiries of your Correspondent, S. M.* relative to the mortality and preservation of leeches; a subject of many other apparently trifing appendages to Medical Science, that not only appears to be little understood, but very much neglected. Very many animals, and indeed almost every living creature, has become more or less the object of the inquiry of ingenious Naturalists; but that which relates to this useful and (I may justly say) valuable reptile, the leech, appears, so far as I am acquainted, to be very much neglected.

To trace its first introduction into medical practice from the earliest period of time, with its natural history, preservation, and management, and to compare its effects with those of venæsection, cupping, blistering, scarification, caustication, cauterization, and various other external means, used for the cure and alleviation of different complaints and diseases; together with what relates to their action and effects produced on the animal system, locally or generally, would, undoubtedly, be of no small acquisition and importance to Medicine. That such an inquiry is not unworthy the attention of some of your learned and ingenious Correspondents, whose leisure, ability, and inclination might induce them to prosecute so useful and necessary a subject, there can be but little doubt.

Your Correspondent, S. M. speaks of the great mortality that happened among his, and other gentlemens' leeches, nearly at one and the same time; and as his paper is dated in the month of August, and these deaths seemed to occur in the preceding months, it is by no means improbable, that such an accident may have proceeded from the heat of the sun, or degree of temperature in which they had been kept. That it has frequently been the case with leeches kept in such situations in summer, I have often known to occur, and sometimes, though not frequently, in very severe winters. Whatever difference there may be in the nature and properties of the water in which they are to be kept, seems to be a matter of dispute; Mr. Whitlam† prefers river to spring water, and your Correspondent S. M. and he

* Medical and Physical Journal, No. 67, Vol. xii. p. 220.

† Ibid. No. 68, Vol. xii.

he are of opinion it should be renewed daily. The former gentleman also recommends that this water, (but for what reasons he has not assigned) previous to its use, should be kept in a cistern two or three days; and in cold weather, that it should be in a tepid state. Mr. Ring says,* "that the wholesale dealers in leeches keep them in spring water during the severity of winter, because it is then warmer than river water; possibly, it may be better than river-water during the summer, because it is then colder, and particularly if the putrefaction in river-water have any share in producing the mortality in question." He also says, "It is obvious that a cool situation should be preferred in summer, and a warm one in winter." These remarks of Mr. Ring, are perfectly consonant to my experience, not only with respect to the spring in preference to that of river water, but also that of the temperature of heat necessary to keep them, which in summer I have deemed not higher than 60°; and in winter not lower than 50° of Fahrenheit's thermometer.

The diurnal change of the water, for which your Correspondent and Mr. Whitlam contend as necessary, though not noticed by Mr. Ring, seems, so far as my observations extend, to be of no essential service. Water, when pure and fresh from a spring or river, is in the Scripture phrase termed *living*; but when deprived of its vitality by putrefaction, or rarefied by heat, becomes dead. This process however, which takes place much sooner in water alone than that in which leeches are kept, even in the same degree of temperature, is a fact that cannot have escaped the notice of those persons, who for any length of time have been in the habit of observing, how much longer water retains its sweetness in which these animals are kept, and also small fish in glass globes.

Perhaps the vital principle of the animal, resident in its natural element, may contribute in no small degree to uphold, or support, for a longer space of time (than it otherwise would do) the vitality of the water; and, *vice versa*, the vitality of the water, when in a due state of temperature, may be said to support the animal.

These old fashioned remarks, which I have hazarded on account of their being easier understood by general readers, (than those of modern chemical phraseology) may perhaps serve to explain the reasons why leeches are more

* Medical and Physical Journal, No. 68, Vol. xii.

more subject to die in vessels containing a great number, and in which there may not be a sufficient proportion of water to each, than of a small number which are kept in a large quantity of water; and also serve to develope, in some measure, the late mortality complained of by your Correspondent S. M. as well as other considerable losses that may have happened to others in similar situations.

Leeches which I have kept, although not more than six or eight together, were usually put in an eight-ounce vial, and have sometimes, even in summer, not had their water changed for some weeks; and in winter I have kept a similar number even to two months or more, yet I have always found the water free from fœtor, although I then changed it; but even this was often done by way of preventing what might not have happened, than perhaps any injury that would have taken place after twice or thrice this space of time had elapsed; for I have known not a few instances in which from one to two or three leeches, kept in a like situation, have remained alive for nine months, or above a year, without a change of water; and this last was not found putrid.

It has not unfrequently happened, that when one, or perhaps two leeches died, that these were such as had a little before been applied; and not being sufficiently disgorged, were then put in a phial with others that had not recently bitten; the blood remaining in them from their bite, being afterwards evacuated in the water, it, in the space of six or seven days, became putrid, but was instantly changed, to prevent the destruction of those remaining. The putrescency of the water in this instance, perhaps, was as much occasioned by the blood as the death of the leeches, which proceeded from their improper treatment, they having been applied by an unskilful person.

Of late, it has been customary for me to keep leeches, immediately after they have bitten, in a separate vial, and to examine them daily, being of opinion that such are more liable to die than those that are fresh, or have bitten some time prior, and are recovered.

No single leech, nor any number that have recently bitten, should be put into a fresh stock, or among such as are in health, after having performed their office; this appears to be more requisite, as, should any of the number die, it will be difficult to determine to what set they appertain, or to what cause their death is to be imputed.

In

In one instance, where a single leech had been kept in water unchanged for more than six months, and in another, where two were in a similar situation, the water of both was not fœtid, but somewhat muddy; this being thrown away, and fresh added, they, in the space of four or five days, were found dead.

From these considerations I have been induced, on changing the water in which leeches are kept, whether for a long or short space of time, except it be once every two or three days that I have occasion to use them (and then I omit this process) to mix one-third, or half as much fresh to the old water; a precaution which, however frivolous it may appear, seems not unnecessary, as the variation or difference between these waters (but on what account I do not pretend to say) most likely occasioned the death of those already noticed.

These occurrences, which seem merely the result more of accidental observation than any designed experiment for the real ascertainment of the facts I have advanced, tend to prove, that the frequent change of water is by no means so absolutely necessary as is supposed; for the proofs already stated appear more, I think, to discourage its frequent use than what has been advanced in its favour. S. M. says, that his, and friend's leeches died, notwithstanding this precaution; and in most instances this has been found to be the case generally, more especially as has been already noticed, where there has been a considerable number crowded together in perhaps too small a portion of water, added most probably to that of a heat of too great a temperature.

The subject next for our consideration is, that of their application, management, and the treatment posterior to the performance of their office. These being of more real importance than superficial, self-sufficient, or inattentive observers may deem necessary, will, I am persuaded, be considered in a very different light by those who have experienced the difficulties, accidents, and disappointments, with which it is not unfrequently attended.

A wine glass, of a larger or smaller circumference, and proportionate to the surface, or part, to which one or more of these animals are to be applied, seems much more convenient than the fingers; and that not only with a view of observing their motions, circumscribing their limits, retaining them in their proper place assigned for their bite, but it also tends to support them when *filling*, and thereby prevents prevents their separating from the parts sooner than they otherwise would do.

To confine them on the temples, pubes, groin, scrotum, knee, or elbow joints, eye-lids when tumefied from blows producing extravasation, face, neck, and certain tumours of a circumscribed magnitude, as well as the limbs of children, &c. a smaller glass will not only be necessary, but the degree of pressure should be such as to prevent their escape, and so managed as not to give pain.

Prior to their being put on, the parts should be cleaned from such applications as have been previously used, with a sponge dipped in soap-lather, and wiped dry. This precaution, though sometimes neglected, seems absolutely necessary, as the effects of some remedies* not only prevent their biting, but to my certain knowledge have almost instantly destroyed them.

On being applied, they sometimes lie long in a torpid state; and at others, they recede, will not bite, or even touch the part, but tenaciously adhere to the top of the glass; on removing them, and applying others, *they* have immediately fixed, and on their falling off after being glutted, those which before refused have afterwards bitten, no doubt from the effects produced on them by the blood, with which they are in contact. To ascertain this fact, I have at times punctured my hand on its upper surface with a lancet, and on rubbing the parts with this blood, frequently succeeded, when for a long time they refused biting, after a small portion of sugar and water, and even milk alone, or mixed with the same, had failed. In short, so much patience, as well as dexterity, is requisite in the management of these capricious, or rather irritable animals, that it may, notwithstanding the apparent simplicity of such an operation, be considered as an art.

Some instances have occurred where they had bitten and remained on the part but for a short space of time, so as to have got but little blood; by dint of patiently retaining them on the part, I have found that they fell to it again, and performed well.

In cases where the use of leeches is urgent, it seems expedient (if they can be procured) to have them not only large ones, but where a number are required to be applied, to be prepared with ten or a dozen; by these means, we have

* Such as volatile liniment, camphorated spirits, ol. terebinth. solutio sal. ammon. &c. &c.

have not only a choice, as some will, and others will not bite, but a greater certainty of effecting our purpose more completely.

It has nevertheless happened not a few times, when I have had occasion to use them in the evening after sun-set, that is, where I used candle-light, although I have sometimes tried half a dozen separately, or two at one time, that they would not bite; moreover, I have also remarked, that in cloudy, or rainy weather, particularly when the barometer was low, they were equally averse.*

These animals, as I have sometimes experienced, do not, and indeed will not, bite on places covered with hair, more particularly on the scalp, where it is strong, and thick set; for admitting this part to be close shaved, I have found the sharp points of the incised hairs so greatly to annoy them, as to prevent their fixing; but on the temples, the hair being of a thinner texture, or less thick, by close shaving, not only a larger space has been procured for their range, but they have more readily bitten. In like manner, if the hair is close shaved from the pubes, groin, scrotum, arm-pits, &c. for reasons already named, they will more readily bite than they otherwise could have done.

Exclusive of what has already been said, there still remains some other remarks necessary to be noticed, which is the situation of the patient's body previous to, or at the time of their application; this should be in a horizontal posture, when applied to the belly, sides, breast, or breasts, (these last, in women, may be elevated, and also the scrotum or testes). But when applied to parts appertaining to the head, as the temples, face or neck, a sitting posture will answer the purpose by reclining it in whatever posture it may be suitable. The upper and lower extremities, with the posterior parts of the body, to which last they are but seldom applied, require no particular directions, but may be managed according to circumstances; those regarding the mouth, such as the gums, lips, or inside of the cheeks, may be left to the prudence and discretion of the operator, or operators. In these last complaints, it however appears

* That leeches are influenced by the state of the weather, there can be no doubt; and on this account many persons have considered them as tolerably accurate barometers; be this as it may, my experience has not yet furnished me with a sufficient number of observations to ascertain this fact with strict accuracy.

appears to me, that their use should be laid aside; for whatever advantage may be expected to be derived from their application in such cases, they certainly are not worthy the attention of the medical adviser, operator, or that of the perplexity, uneasiness, and risque incurred by the patient; and this more especially as it is safer to use the lancet where gum-boils or abscesses are to be opened; or if this is not necessary, scarifications or incisions.

I should have noticed, that when the leeches are firmly fixed on the part or parts, and in the act of *suction*, they ought by no means (which not unfrequently happens from the awkwardness of the patient, or person holding the glass) to be disturbed; this ought to be so managed as to support them when fixed, till they are glutted; the glass being reclined so as to admit air; and the operator, while in the act of performing his office, should, to prevent his arm being wearied, rest it on something solid.

Supposing however it should happen, which it would not, if the operator was not a mere bungler, he having the leeches in the glass to see what is going on; I say, were they to bite, or fix on improper places, or what is full as bad, on his fingers, or hand, common salt being always ready, (he should be careful not to pull at the animal lest he destroy it, and perhaps ineffectually put the patient or himself to unnecessary pain) a little should be put on its mouth, which will instantly cause it to fall off safely.

Our next inquiry relates to their treatment after having bitten, the nice management of which, is of the utmost importance for their future preservation; and this is the more to be regarded when we reflect, that after having done their office, they are by many considered of no further use.

By some, their death has been ascribed to what they term, the poisonous effects of the blood, or other fluids imbibed; and this opinion has become stronger in proportion to the greater or less distance of the time of their dying when separated from the parts bitten. If, e. g. as is usually the case, they are thrown on a large quantity of salt provided for their purgation, their immediate death, which is the necessary consequence, is ascribed to the cause above mentioned, and is also considered in the same light, should they die in a longer or shorter time after being put into water; but if they fortunately escape death from the more skilful management by the salt not coming in contact with other parts of their body, excepting their

6 mouths, mouths, the marvellous opinion of the poison received, vanishes.

Whatever may be the notion of the poisonous effects of blood sucked by leeches, so as to occasion their death, is to me a mere *chimera;* I do not think they can be injured by it, provided they are properly managed after performing their office, and this not only from reasoning, but my extensive experience convinces me of its fallacy.

Neither do I believe that a leech or leeches, which may have been previously applied to patients in the small-pox, measles, scarlet fever, venereal buboes, or other affections of this sort, or to cancerous, venereal, or phagadenic ulcers, bite of a mad dog, or any other specific disease whatever, will or can communicate a similar affection; and this no more than I ever believed that the matter taken for inoculation from the small-pox of a child, labouring at the time under scrophula or lues, could communicate those diseases. As for any other mode of treating them than that with common salt, I profess myself totally unacquainted; but as this has answered completely to my satisfaction, and needs only to be used with caution, I shall proceed to relate in what manner it is to be used. Having prepared a large shallow clean plate, and some salt by itself, together with a bason half full of fresh water, I put the leech or leeches thereon, after they have bitten. I then take a small portion of salt, equal to a moderate pinch of snuff, compressed between my thumb and fore finger, to make it fine, and place it in contact with the mouth of the animal. I carefully avoid at the same time, touching with it any other parts of its body, which, should this happen, may be easily pushed gently off with the fingers. This causes it to contract itself, should it not immediately disgorge, into a thick short round form, while at the same time it projects, and turns up its mouth into a small, tapering appearance, resembling a proboscis, and in this state will often remain for some seconds, in a sort of torpor, till at length it recovers by disgorging a small quantity, and at other times does not. The salt, which I afterward increase a little in quantity, is then so put on the plate, that the animal in its progressive motion may come as nearly as possible in contract with it, by its mouth, no other parts being allowed to touch the salt, to prevent the injury resulting from its application, which, should this happen, will more or less damage it, by corrugating, or blistering its body. This mode of procedure, though slow, is not altogether unamusing, and it is curious to perceive how it avoids avoids the salt on slightly touching it with its mouth, when if it should come in contact beneath this part, it does not appear to be disturbed; but should it firmly fix its mouth on the salt, which is often the case, it is sure to vomit most plentifully, sometimes in a small thready stream, and at others in small gulps; the blood, as may be naturally expected, is at first of a moderate red colour, but on mixing with the salt, a beautiful scarlet hue diffuses itself, as it were, *quaquaversum*, from the centre to the circumference. In this manner, it is necessary to proceed by alternately placing the salt to its mouth, till by reiterated disgorgements the animal has emptied itself, which may be known from its being reduced to the size it appeared prior to its application, and from its ceasing to vomit on coming again in contact with the salt; but as the dose of the salt may be carried to too great an excess by its frequent repetitions, and has happened to be the death of two of my vermicular patients, a third, after remaining a long time in a state of torpor, recovered, it behoves us to act with no small caution; and as a proof of having carried this evacuating plan too far, the leech, when sufficiently purified from its blood, not only appears to be reduced to its natural size, but is apt from being before this, brisk and lively, to become, when plied with too much salt, inactive, and turns on its back. It is then high time to throw it into the bason of fresh water, and if it is free from injury, it will immediately frisk and play about, although I have known them, after remaining for some time in a state of torpor, ultimately recover. Such of these leeches as have gone through the processess we have described, and appear to be perfectly recovered, should be kept separate from those that are sickly, or in a state of vital suspension; this is the more necessary, as in case of death, they will be apt (as has already been hinted) to prove destructive to those which are in health.

I ought to have stated, that where much blood was evacuated on the plate, from one or two leeches falling off together or separately, it should be afterwards cleaned with a wet sponge, in order to admit others, as well as to avoid the confusion and embarrassment by its quantity and disagreeable tenacity, enveloping and adhering to the leeches, as well as with a design to observe their motions, when under the evacuating plan; and I must also remark, that placing a little salt occasionally at the verge, or extreme circular boundary of the diffused blood, in which they are surrounded, this salt being diluted, has the advantage vantage of operating less forcibly on every part with which it comes in contact, and is the less liable to do them any material injury. Separate plates, or rather cleaning one plate, after each leech has been purified, seems necessary from what has been said.

Leeches, managed in the cautious manner we have observed, most assuredly will survive after their operation, be they ever so frequently applied; and it is remarkable, that such as are in the habit of biting, are preferable to those that are fresh or untried, as they more greedily fasten themselves on the parts to which they are applied. The larger they are the better; the middle size require more in number to equal the effects of but a few of the former, while the small ones, even in diseases of children, appear to be but of very little use, excepting in some cases of extravasation of blood not deeply seated; and these last, even from the gentle treatment recommended after biting, are much less liable to survive.

From what has been said, it evidently appears, that the consumption or rather destruction of leeches, proceeds perhaps as much from the barbarous murders injudiciously committed on them after having performed their office, as that of keeping or preserving them for mere sale for the consumers. And it may be taken for granted, that these last persons, from the sordid avarice of the retail dealers, who have lately experienced the sweets of their enormous profits,* will hereafter, by their keeping up an artificial scarcity, still continue (if not checked) their impositions.

It cannot therefore be doubted, that should these animals be carefully preserved, which by skilful management after their operation (from the rules prescribed) they are most likely to be, their scarcity and enormous price will not only be lessened, but their use more generally extended, and that among the inferior class of people, which at their present exorbitant price, seems almost impossible.

As a positive and convincing proof of what I have just now advanced, exclusive of what I have experienced generally, I must beg leave to remark, that being under the necessity myself, of using leeches in a complaint requiring their reiterated application for more than seven months, and

* Your Correspondent, S. M. says, that in February and March, this present year, they could not be purchased in Covent-Garden Market for less then 3s. 6d. each. At present, August 6, they fetch 8 or 9s. per dozen; less than twelve years ago, he bought them for 2s. 6d. or 3s. per hundred. Med. and Phys. Journal, No. 67, Vol. xii.

and not being able to procure more than ten of a tolerably large size, I applied five, alternately, for at least fifty times, in the space above mentioned; of these, two were lost, a third was given to a patient, which was killed by mal-treatment, a fourth died, and the rest remain in perfect health.

To point out more clearly the important advantages resulting from such laudable industry in the preservation of these useful animals, I have made the following estimate: And though, according to the careless treatment generally used, one leech seldom bites more than once; yet, to give a fair and reasonable statement, I have allowed such to bite twice.

| | |
|---|---|
| Number of leeches used — — — — | 10 |
| Applied alternately, five each time, to the number of | 50 |
| Number of bites for the whole, amount to — | 250 |
| Number of bites for each, considering their alternate application — — — — — — | 25 |
| Number required according to the general treatment to perform the same number of bites, allowing each to bite twice — — — — — — | 125 |

| | | | |
|---|---|---|---|
| This number, at 3s. 6d. each, as lately sold at Covent-Garden Market, amounts to the sum of | 21 | 17 | 6 |
| Ten, at the same price, will amount to — | 1 | 15 | 0 |
| Money saved, £. | 20 | 2 | 6 |

In this part of the world, they are sold at a more reduced price, about 9s. per dozen; this, however variable, produces no alteration in the comparative difference, but leaves the money saved proportionably the same.

If, in the instance we have related, this œconomical practice appears so highly beneficial, its general adoption cannot but be productive of immense advantages, as the great benefit derivable from the *œconomy of life* in these hitherto neglected animals, will become a principal means of preventing their future scarcity, which, in not a few instances, where their use is absolutely necessary, cannot easily be replaced by any other means capable of producing similar effects.

*Sunderland, Nov. 5, 1804.*

